# A postpartum intervention for vaccination promotion by midwives using motivational interviews reduces mothers’ vaccine hesitancy, south-eastern France, 2021 to 2022: a randomised controlled trial

**DOI:** 10.2807/1560-7917.ES.2023.28.38.2200819

**Published:** 2023-09-21

**Authors:** Pierre Verger, Chloé Cogordan, Lisa Fressard, Virginie Gosselin, Xavier Donato, Magalie Biferi, Valérie Verlomme, Pierre Sonnier, Hervé Meur, Philippe Malfait, Patrick Berthiaume, Lauriane Ramalli, Arnaud Gagneur

**Affiliations:** 1 ORS PACA, Southeastern Health Regional Observatory, Marseille, France; 2 Centre de recherche du CHUS, Sherbrooke, Québec, Canada; 3 Maternity ward, Saint-Joseph Hospital, Marseille, France; 4 Maternity ward, Sainte-Musse Hospital, Toulon, France; 5 Comité Régional d’Education pour la Santé Provence-Alpes-Côte-D’Azur, Marseille, France; 6 Agence Régionale de Santé Provence-Alpes-Côte-D’Azur, Marseille, France; 7 Santé publique France (French National Public Health Agency), Marseille, France; 8 Les formations perspective santé Inc., Montréal, Québec, Canada

**Keywords:** Childhood vaccination, Vaccine hesitancy, Vaccine intention, Motivational interviewing, randomised controlled trial

## Abstract

**Background:**

Despite childhood vaccine mandates imposed in 2018 in France, parental vaccine hesitancy (VH) remains frequent. Interventions in Quebec, Canada, applying motivational interviewing (MI) techniques have successfully reduced parents’ VH for childhood immunisations.

**Aim:**

To determine whether MI intervention for mothers in maternity wards in the days after birth in France could significantly reduce VH, increase intentions to vaccinate (VI) their child at 2 months and reduce VH social inequalities.

**Methods:**

We conducted a parallel-arm multicentre randomised controlled trial from November 2021 to April 2022 to compare impacts of MI performed by MI-trained midwives (intervention) vs a vaccination leaflet (control). We included 733 mothers from two maternity hospitals in south-eastern France, randomly assigned either arm. The validated Parents Attitudes about Childhood Vaccines questionnaire was used before and after MI or leaflet to assess mothers’ VH (13 items, 0–100 score) and VI (1 item, 1–10 score). Difference-in-difference (D-I-D) models were used to estimate net impact of MI vs leaflet for the entire sample and stratified by VH and education level.

**Results:**

Motivational interview intervention reduced mothers' VH score by 33% (p < 0.0001) and increased VI by 8% (p < 0.0001); the effect was largest for the highest initial VH levels. D-I-D analyses estimated net VH decrease at 5.8/100 points (p = 0.007) and net VI increase at 0.6/10 points (p = 0.005). Net VH decrease was highest for high initial VH levels and low education levels.

**Conclusions:**

Our results show positive effects of MI intervention, and means of its implementation should be investigated in France.

**Key public health message**

**What did you want to address in this study?**

In France, 11 infant vaccines are required for entry to daycare and school, yet vaccine hesitancy is still frequent among parents. We wanted to evaluate whether motivational interviewing by midwives, carried out in maternity wards with mothers in the days after giving birth, could impact vaccine hesitancy.

**What have we learnt from this study?**

We found that motivational interviewing of mothers in maternity wards significantly reduced their vaccine hesitancy. The women who were initially the most hesitant to vaccinate their newborns were the most impacted by the interviews. We also found that a reduction in vaccine hesitancy was greatest for mothers with low education levels.

**What are the implications of your findings for public health?**

Our results, combined with high participant satisfaction with the intervention overall and for several of its specific aspects, suggest that motivational interview can be applied in maternity wards in the days after birth and is an effective strategy to reduce vaccine hesitancy and improve mothers’ intention to vaccinate their newborn child.

## Introduction

Vaccination is among the most effective ways to avoid mortality and morbidity from vaccine-preventable diseases. Its ability to provide collective protection, however, depends on high vaccine coverage (VC) rates (≥ 95%), which many countries do not reach [1], partly due to vaccine hesitancy (VH) [2]. In France, where childhood VC was suboptimal in the 2010s [3], a 2016 study showed a VH prevalence of 40% among parents [4]. Furthermore, social inequalities in childhood VC persist in high-income countries, in some cases to the detriment of the people who belong to socially deprived population groups [5]. To address these suboptimal childhood VC rates (e.g. 70% for children aged 24 months for meningitis C in 2016) [6], in 2017 the French Ministry of Health expanded vaccination requirements for young children's admission to daycare and school from three to 11 specific diseases [7]. Although this measure increased childhood VC [8], overall coverage still falls short of the 95% target for some vaccines [9]. Data suggest that delays between the officially recommended and actual age at vaccination may persist, and be more pronounced in people belonging to deprived populations (data not shown); moreover, this measure has met reluctance from a quarter of parents of young children [10] and may have even reinforced VH [11]. Personalised information and/or education programmes are thus needed, but evidence that such interventions improve childhood VC and parents' intention to vaccinate their children (VI), especially when implemented top-down, is at best moderate [12]. Thus, it is important to seek new approaches for providing parents with information that meets their needs [13].

The motivational interview (MI) [14] is a collaborative conversational style that reinforces a person's own motivation and commitment to behaviour change and involves a partnership relationship with the professional. MI was successfully tested in multiple domains related to health behaviour change [14]. In Quebec, the MI approach was adapted to childhood vaccination to understand reasons behind parents‘ hesitation to have their children vaccinated and guide them towards the aim of changing behaviour. It was tested post-partum with parents in maternity wards (PromoVac and PromoVaQ studies) [15-20]. It applies the following principles: (i) establish a trusted relationship with parents, (ii) understand the specific reasons for their hesitancy (iii) deliver information they request and (iv) try to direct the conversation towards a more favourable position towards vaccination. In these studies, MI implementation led to a 40% decrease in mothers’ VH [20] and a 6-percentage point increase in infant VC 7 months after the intervention [16]. Quebec therefore began extending this approach to all maternity wards in 2017 (EMMIE programme: Motivational interviewing in maternity wards for child immunisation) [21].

To determine whether these results could be attained in the French context, we conducted a randomised controlled trial (RCT) from November 2021 to April 2022 in two maternity wards in south-eastern France. The primary objective was to provide evidence that, given the French setting of childhood vaccination requirements expansion, a post-partum educational strategy based on MI offered to mothers can significantly reduce their VH and increase their intentions to have their child vaccinated, compared with standard care. Two secondary objectives were to (i) test the differential impact of MI on VH and VI according to social status and (ii) evaluate mothers’ acceptance of and satisfaction with this approach. 

## Methods

### Trial design

From November 2021 to April 2022, we implemented a parallel-arm, multicentre RCT with individual randomisation, comparing the impact assessed by a pre-post questionnaire of MI intervention versus standard care (educational leaflet about vaccination) as the control (ClinicalTrials.gov: MOTIVAC-MATER-Confiance, number NCT05093452). This article presents the results of the comparisons just after the MI or leaflet distribution; further results of the RCT covering a 7-month follow-up will follow this initial report.

### Study setting and participants

Participants were mothers, included either alone or with their spouse/partner, recruited in the maternity wards of two general hospitals in south-eastern France: Saint-Joseph Hospital (781 beds, Marseille, ca 5,000 deliveries annually) serving a mixed and less-affluent population, and Sainte-Musse Hospital (736 beds, Toulon, ca 3,000 deliveries annually) serving a more affluent population [22]. In 2020, the average length of stay in French maternity wards was ca 3.7 days but varied widely [23]. The prevalence of VH in south-eastern France is higher than elsewhere in France, as shown during the COVID-19 pandemic [24] and early in surveys of healthcare providers [25]. 

Eligibility criteria for participants were: age 18 years and older, residence in one of the two districts of the study area at the time of delivery and the ability to read and speak French. For reasons of feasibility, mothers were excluded if they were COVID-19-positive, if they or their newborn child had a severe medical condition, or if they were discharged within 24 h of delivery for any other reason.

### Sample size

We estimated the required number of mothers/couples needed as follows: (i) we aimed to be able to demonstrate a 20% difference-in-difference (D-I-D) in mean VH scores between the two groups (i.e. at least 30% reduction in the MI group and 10% reduction in the control group at two points in time: just after the MI or leaflet distribution and at follow-up); (ii) expected 20% loss-to-follow-up rate at follow-up, based on previous interventions [26,27]; (iii) 5% risk of a type I (α) error and 80% statistical power; (iv) mean initial VH score of 40% (average VH score among parents of children in France) and coefficient of variation of 0.80. After 1,000 data simulations, we concluded that 550 participants were required in each group.

### Procedure and study arms

Each workday, three midwives volunteering for the study (two half-time at Sainte-Musse and one full-time at Saint-Joseph) visited mothers who had given birth the previous day, identified from each maternity ward's delivery register. The midwives presented the objectives of the study to the mothers and their partners (if present), checked the inclusion/exclusion criteria and collected their consent. 

Midwives registered mothers who agreed to participate on a tablet computer containing software that automatically randomly assigned participants to the groups with a 1:1 ratio, in blocks of eight, after completing the first questionnaire, to avoid bias.

#### Intervention arm: midwife motivational interview training

Midwives underwent a two-part training over 2 months to acquire skills to perform MI. In September 2021, midwives followed a 5.5-h e-learning programme [28], which included topics such as vaccination principles, French vaccine policy and specific modules for each childhood vaccine. They also received a 66-page guide summarising vaccination information useful for answering parents’ questions [29]. At the beginning of October 2021, two study authors (MI specialists) trained the midwives during a 2.5-day face-to-face workshop about MI's theoretical foundations, its adaptation to vaccination and role-playing exercises. The trained midwives then put this approach into practice with mothers during a pilot phase the following month. During this time, midwives underwent a 2-h group debriefing and two individual conversations with one of the MI specialists based on a MI audio recording by a trained midwife in order to address potential difficulties in practicing MI. The study period where questionnaires (T0 and T1) were conducted began at the end of the pilot phase, from November 2021. The midwives performed MI individually with participants in sessions that lasted 10 to 30 min, as needed, applying the following principles: (i) establish, as the initial objective, a trust relationship by listening carefully and without judgement to the mothers’ and, where applicable, partners’ concerns, without trying to correct or argue against certain information; (ii) understand the specific reasons for their hesitation to ascertain what information would improve their perception of vaccination's importance; (iii) deliver this information with their agreement, to support their personal choice; and (iv) respect their personal autonomy and try to direct the conversation towards a more favourable position towards vaccination, while encouraging partnership and avoiding discord.

#### Control arm: leaflet

Midwives distributed a four-page leaflet about vaccination of newborns to participants randomised to the control group [30]. This leaflet, expected to have no or little effect on mothers’ VH [18], was provided to estimate the Hawthorne effect, i.e. the bias related to a change in the behaviour of both the participant and staff because of the knowledge that they were being observed or to desirability concerns [31].

### Data collection and outcomes

We collected data through two self-administered questionnaires, one before the MI or leaflet distribution (T0, pre-questionnaire, 34 items) and a second afterwards (T1, post-questionnaire, 35 items). They were completed by study participants during the post-partum stay at the maternity ward; 80% of the participants completed both on the same day.

Both questionnaires can be found in the Supplementary Material S9. The T0 questionnaire collected respondents' demographic and socioeconomic characteristics, which included age (continuous), living with a partner (yes/no), birth rank of the newborn (continuous), education level (high school or below/at least some post-secondary education), and perceived financial situation (level of security in six categories: cannot manage without debts, cannot manage easily, have to be careful, only fair, OK, comfortable). Education level and perceived financial situation (the latter was grouped into two categories for the analyses: cannot manage without debts or easily (‘insecure’) vs the other four categories (‘not insecure’)) were used each individually as proxies for social deprivation level. The survey also collected mothers' own vaccination status against influenza during pregnancy (yes/no).

Using the T0 and T1 questionnaires, we collected data related to the two primary outcomes: (i) VH assessed with a modified version of the Parents' Attitudes about Childhood Vaccines (PACV) [32] validated questionnaire (13 items, see Supplementary Material S1 for PACV adaptation) used in the PromoVac and PromoVaQ studies [15,18-20] and (ii) VI (i.e. intention to vaccinate their infant) at 2 months assessed with the following item, also used in those studies: How sure are you that you will vaccinate your baby at 2 months of age? (on a scale from 1 = 'not certain at all' to 10 = 'absolutely sure').

Finally, we also collected data on a secondary outcome, based on results from the T1 questionnaire, related to mothers’ acceptance of/satisfaction with the MI/leaflet (wording differed slightly for the MI and the leaflet).

### Statistical methods

A VH score was constructed at T0 and T1 by summing the 13 PACV items by Opel's approach [32]: 2 points for hesitant responses, 1 point for ‘not sure or don’t know’ responses and 0 points for non-hesitant responses. Then a linear transformation was applied to convert the sum into a 0–100 scale [32]. Three categories of VH were defined as low (score 0 – < 30), moderate (30 – < 50) or high (50 or more) [20].

We used chi-squared tests (categorical variables) and Wilcoxon rank-sum tests (continuous scores) for baseline comparisons. For comparing the impact of the MI-based intervention with that of the control on both VH and VI scores, we computed mixed models with random intercepts to create D-I-D models. These allow changes over time (pre- and post-intervention) of an outcome to be compared between two groups, while taking into account the repeated nature of the data [33,34]. The details of the equation can be found in the Supplementary Material S2. We first estimated overall impact of MI on outcomes compared with the leaflet. Then we stratified for the initial VH level to assess the effects of MI and leaflet by initial VH level. To test the differential impact of MI on VH and VI scores by social deprivation level (secondary objective), we stratified the analyses by education level and perceived financial situation, used as proxies for social deprivation level. Given the similarity of their results, we present only those for education level in the Results section.

The overall D-I-D models were performed without covariates and then adjusted for maternity ward, age, education level, perceived financial situation, birth rank of the newborn and influenza vaccination during pregnancy. Analyses were performed by intention-to-treat (ITT): all patients who agreed to participate and completed the pre-questionnaire (T0) were included, with missing post-questionnaire (T1) data handled by the mixed model without imputation [35]. Per protocol (PP) sensitivity analyses were conducted by excluding patients who did not respond to the post-intervention questionnaire. Analyses used SAS 9.4 (SAS Institute Inc.), with statistical significance set at 0.05.

## Results

**Figure 1:**
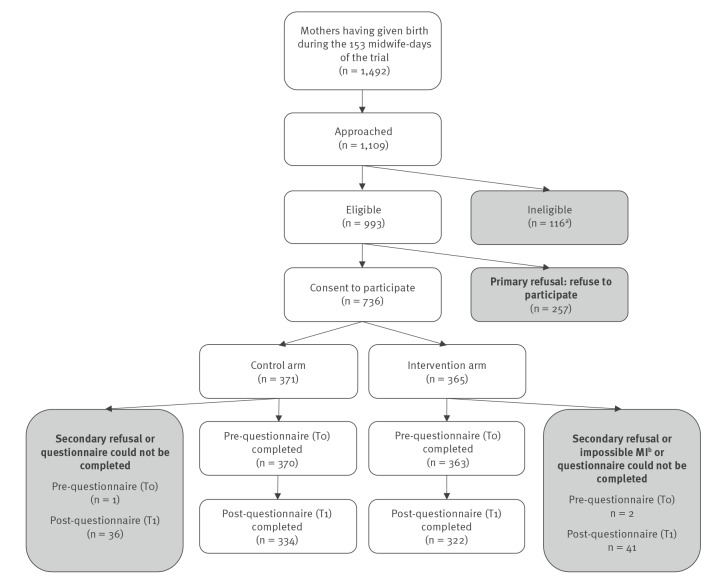
Flowchart of inclusion criteria, south-eastern France, November 2021–April 2022^a^

During the period of midwives’ activity in the course of the survey, 1,492 mothers gave birth and 1,109 (74.3%) could be approached (Figure 1). The other 383 were excluded since they either tested positive for COVID-19 (n = 64), or mother and/or newborn had a serious medical condition, or were not approached because they were asleep at each attempted midwife visit or could not be visited because of lack of time. Among those approached, 993/1,109 (89.5%) were eligible and 736 (74.1%) of them agreed to participate. The 257 who refused cited reasons including fatigue (n = 145), lack of time (n = 57) or lack of interest in vaccination (n = 53) (several reasons possible). 

MI: motivational interview.

^a^ Ineligible: age under 18 years, residence outside the two districts of the study area at the time of delivery, inability to read and speak French, COVID-19-positive at the time of delivery, severe medical condition of the newborn and/or mother preventing participation and/or requiring transfer to a neonatal unit/another maternity hospital or discharged within 24 h of delivery for any other reason.

^b^ Impossible MI: the MI could not be carried out because the mother was not present in her room or was not available when the midwife visited her.

During the study period, 1,492 mothers gave birth and 1,109 (74.3%) could be approached. All participants who completed the pre-questionnaire (T0) were included in intention-to-treat (ITT) analyses, while only those who completed both pre- and post-questionnaires (T1) were included in the per-protocol (PP) sensitivity analyses.

Participants were randomly assigned to the control group (leaflet, n = 371) and the intervention group (MI, n = 365). In the control group, 370 participants (99.7%) completed the T0 questionnaire and 334 (90.0%) the T1; these figures in the intervention group were 363 (99.5%) and 322 (88.2%), respectively. In total, 733 mothers were included in the ITT analyses and 656 in the PP analyses.

### Baseline data

The percentages of participants who completed only the T0 questionnaire were similar in both groups (MI: 41/365 (11.2%); control: 36/370 (9.7%); p = 0.49), but dropouts were observed more frequently from Saint-Joseph (22.1%) than Sainte-Musse (1.7%; p < 0.0001, see Supplementary Material S3, Table S1 for the distribution of the groups across hospitals).

Participants in the two groups did not differ regarding sociodemographic characteristics (Table) or mean initial VH score, which was moderate (34.0/100 ± 21). Initial VH distribution by arm (intervention/control) was low (43.3%/42.7%), moderate (29.8%/32.7%) and high (27.0%/24.6%). Nonetheless, T0 scores for intention to have the infant vaccinated at 2 months of age were higher in the control group (8.7/10) than in the intervention group (8.3/10, p = 0.02).

**Table:** Characteristics of study participants by randomisation group, south-eastern France, November 2021–April 2022 (n = 733)

Characteristics	Randomisation group				All (n = 733)		p value
	MI-based intervention (n = 363)		Leaflet (n = 370)				
	n	%	n	%	n	%	
Age of mother (years)							
All, mean (SD)	30.7 (4.9)		31.2 (5.0)		31.0 (5.0)		0.41
18–24	38	10.5	27	7.3	65	8.9	0.32
25–29	106	29.2	112	30.2	218	29.7	
30–34	139	38.3	135	36.5	274	37.4	
≥ 35	80	22.0	96	26.0	176	24.0	
Lives with a partner^a^							
Yes	322	89.0	345	93.8	667	91.4	0.07
No	28	7.7	16	4.3	44	6.0	
Don't know/refuse to answer	12	3.3	7	1.9	19	2.6	
Birth rank of the newborn							
1	195	53.7	178	48.1	373	50.9	0.13
2 or more	168	46.3	192	51.9	360	49.1	
Education level^b^							
Low: equivalent to high school or lower	139	38.4	121	32.8	260	35.6	0.27
High: at least some post-secondary education	213	58.8	235	63.7	448	61.3	
Don't know/refuse to answer	10	2.8	13	3.5	23	3.1	
Perceived financial situation^a^							
Insecure	116	32.0	102	27.7	218	29.9	0.14
Not insecure	219	60.5	247	67.1	466	63.8	
Don't know/refuse to answer	27	7.5	19	5.2	46	6.3	
Vaccinated against seasonal influenza during pregnancy^b^							
Yes	42	11.6	42	11.4	84	11.5	0.93
No	315	87.0	323	87.5	638	87.3	
Don't know/refuse to answer	5	1.4	4	1.1	9	1.2	
Maternity ward							
Sainte-Musse	207	57.0	209	56.5	416	56.8	0.88
Saint-Joseph	156	43.0	161	43.5	317	43.3	
Agree to be contacted again for another questionnaire							
Yes	285	78.5	277	74.9	562	76.7	0.44
No	73	20.1	85	23.0	158	21. 6	
Don't know/refuse to answer	5	1.4	8	2.1	13	1.8	
Vaccination							
Initial vaccine hesitancy score (0–100), mean (SD)	34.8 (21.1)		33.2 (20.1)		34.0 (20.6)		0.43
Initial score of intention to vaccinate one’s infant at 2 months of age^c^ (1–10), mean (SD)	8.3 (2.1)		8.7 (2.0)		8.5 (2.0)		0.02*
Post-intervention questionnaire (T1)							
Completed	322	88.7	334	90.3	656	89.5	0.49
Missing	41	11.3	36	9.7	77	10.5	

MI: motivational interviewing; SD: standard deviation.

^a^ Missing values (n = 3).

^b^ Missing values (n = 2).

^c^ Missing values (n = 11).

Because of rounding, the sum of the percentages may not equal 100%. Chi-squared tests were used for categorical variables and Wilcoxon rank sum tests were used for continuous variables.

An asterisk marks a p value ≤ 0.05, which was considered significant.

### Differences in pre-post vaccine hesitancy changes

#### Overall difference

The control group's average VH score was 33.2/100 at T0 and 27.6/100 at T1, a 16.7% decrease (−5.5 points, p = 0.0002). In the MI group, the corresponding scores were 34.8/100 at T0 and 23.4/100 at T1, a 32.7% decrease (−11.4 points, p < 0.0001). The estimated D-I-D effect without covariables, that is, the net T1 − T0 difference of the VH scores between the MI and control groups was −5.8 points (p = 0.01); adding covariables to the overall model produced a similar result (D-I-D effect: −5.0 points, p = 0.02, Supplementary Material S4, Table S2 shows the detailed results of the D-I-D models with and without covariables).

#### Differences after stratification for initial vaccine hesitancy score

**Figure 2:**
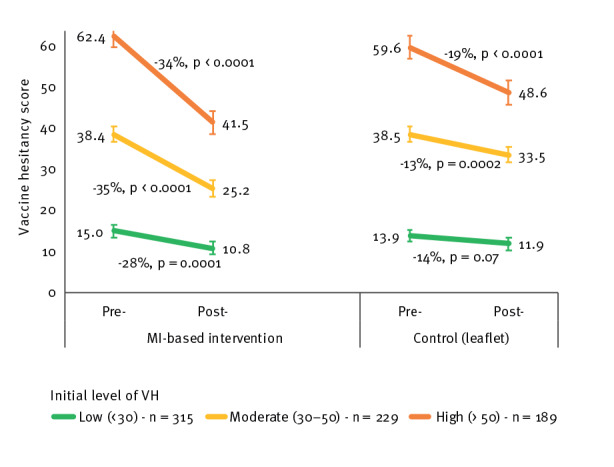
Changes in vaccine hesitancy scores by intervention group according to initial vaccine hesitancy category, south-eastern France, November 2021–April 2022 (n = 733)

D-I-D: difference-in-difference; MI: motivational interview; VH: vaccine hesitancy.

Analyses stratified by initial VH scores showed a decrease in the control group by 18.5% in the high VH category (p < 0.0001) and by 13.0% in the moderate VH category (p = 0.0002, Figure 2). In the MI group, VH decreased by 33.5% in the high and by 34.5% in the moderate VH categories (p < 0.0001). Participants with already low levels of VH at T0 showed a significant 28.1% decrease after MI (p = 0.0001), but not after control intervention (p = 0.07). The D-I-D effect was significant in the high and moderate VH categories (high baseline score: −9.9 points, p = 0.001; moderate: −8.3 points, p < 0.0001) but not the low VH category (−2.3 points, p = 0.14).

Vaccine hesitancy scores were estimated with 95% confidence intervals in the D-I-D model, at T0 and T1, according to group (MI or control) and initial VH category (low: n = 157 (MI) and 158 (control); moderate: n = 108 (MI) and 121 (control); high: n = 98 (MI) and 91(control)). In the high VH category, for example, VH scores in the MI group decreased by 33.5%, from 62.4% to 41.5% (p < 0.0001).

#### Differences after stratification for education level

**Figure 3:**
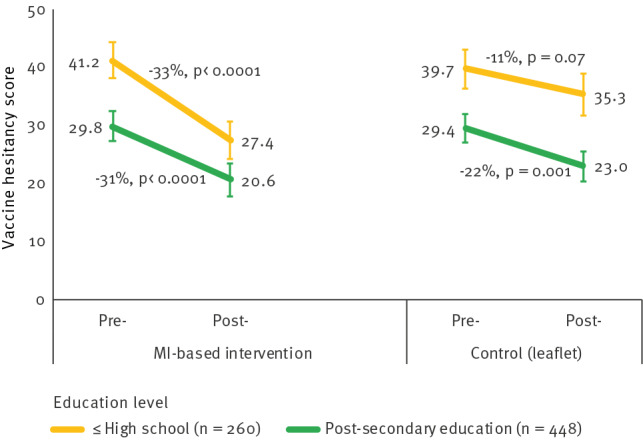
Changes in vaccine hesitancy scores by intervention group according to education levels, south-eastern France, November 2021–April 2022 (n = 708^a^)

D-I-D: difference-in-difference; MI: motivational interview; VH: vaccine hesitancy.

^a^ Some participants (n = 23) did not know about their education level or refused to answer, or data were missing (n = 2).

Participants with a low education level (equivalent to or lower than high school) had higher initial VH scores than those with a high education level (post-secondary education), in both the MI (41.2 vs 29.8/100) and the control (39.7 vs 29.4/100) groups. In the MI group, VH decreased by around one third in both education categories (p < 0.0001) (Figure 3), while in the control group, VH decreased by 21.9% among those with a high education level (p = 0.001) but not significantly among those with a low education level (p = 0.07). The D-I-D effect of MI vs the control intervention on VH scores was significant among participants with a low education level (−9.3 points, p = 0.01) but not among those with a high level (−2.7 points, p = 0.31).

Scores for VH were estimated with 95% confidence intervals in the D-I-D model, at T0 and T1, according to group (MI or control) and education level (≤ high school: n = 139 (MI) and 121 (control); post-secondary education: n = 213 (MI) and 235 (control)). Among the participants with less education, for example, VH scores in the MI group decreased by 33.3%, from 41.2% to 27.4% (p < 0.0001).

### Differences in pre-post changes of vaccination intentions

#### Overall difference

In the control group, the average VI score, i.e. intention to vaccinate one’s infant at 2 months of age, did not increase significantly: from 8.7 to 8.8/10 (p = 0.28). In contrast, the VI score did increase in the MI group: from 8.3 to 9.0/10 (+8.5%, p < 0.0001). The D-I-D effect between MI and control groups was estimated at +0.6 point for the VI score (p = 0.005); adding covariables to the overall model produced the same results (see Supplementary Material S5, Table S4 for the detailed results of the D-I-D models with and without covariables).

#### Differences after stratification for initial vaccine hesitancy score

**Figure 4:**
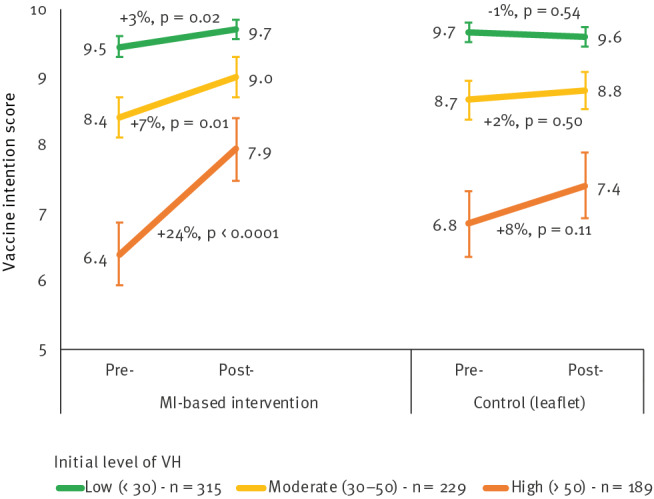
Changes in vaccination intention scores by intervention group according to initial vaccine hesitancy category, south-eastern France, November 2021–April 2022 (n = 733)

D-I-D: difference-in-difference; MI: motivational interview; VH: vaccine hesitancy.

Analyses stratified for the baseline VH score showed that the VI scores were higher when initial VH was lower in both groups (Figure 4). In the MI group, VI scores increased significantly regardless of initial VH score category, with the highest increase (+24.2%, p < 0.0001) in the highest initial VH category. The control group showed no significant increase in any initial VH category. The D-I-D effect of MI on VI scores was significant in the high and low categories of initial VH (high initial score: +1.0 point, p = 0.04; low initial score: +0.3 point, p = 0.04). In the moderate VH category, the difference in the VI change between the two groups was not significant (+0.5 point, p = 0.12).

Vaccination intention scores were estimated with their 95% confidence intervals in the D-I-D model, at T0 and T1, by group (MI or control) and initial VH category (low: n = 157 (MI) and 158 (control); moderate: n = 108 (MI) and 121 (control); high: n = 98 (MI) and 91 (control)).In the low VH category, for example, vaccination intention scores in the MI group increased by 2.6%, from 9.5 to 9.7/10 (p = 0.02).

#### Differences after stratification for education level

**Figure 5:**
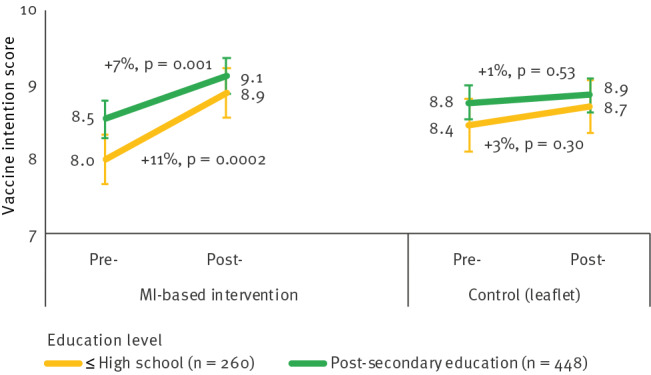
Changes in vaccination intention scores by intervention group and education level, south-eastern France, November 2021–April 2022 (n = 708^a^)

D-I-D: difference-in-difference; MI: motivational interview; VH: vaccine hesitancy.

^a^ Some participants (n = 23) did not know about their education level or refused to answer, or data were missing (n = 2).

Participants with a low education level had initial VI scores lower than those with higher education, in both the MI (8.0 vs 8.5/10) and control (8.4 vs 8.8/10) groups. In the MI group, VI increased by 11.1% among participants with low education levels (p = 0.0002) and by 6.8% in those with a high education level (p = 0.001) (Figure 5). Vaccine intention did not change significantly in the control group, regardless of education level. The D-I-D effect of MI compared with the leaflet on VI scores was positive and significant among participants with a high education level (+0.5 points, p = 0.05) and near the limit of significance among those with less education (+0.6 points, p = 0.07).

Vaccination intention scores were estimated with their 95% confidence intervals in the D-I-D model, at T0 and T1, by group (MI or control) and education level (≤ high school: respectively n = 139 in MI and 121 in control group; at least some post-secondary education: n = 213 and 235). Among participants with less education, for example, vaccination intention scores in the MI group increased by 11.1%, from 8.0 to 8.9/10 (p < 0.0001).

Per protocol analyses are displayed in Supplementary Material S4 Table S3 (VH score) and Supplementary Material S5, Table S5 (VI score). All overall analyses of the entire PP sample yielded similar results. The ITT analyses stratified for perceived financial situation yielded results similar to those for education level as shown in Supplementary Material S6, Figure S1 for the VH score and Supplementary Material S7, Figure S2 for the VI score.

### Satisfaction of participants with motivational interviews

In the MI group, 99.4% (320/322) mothers responded to the satisfaction questionnaire: 95.0% (304/320) reported they appreciated participating (somewhat yes: 56/320 (17.5%); yes: 248/320 (77.5%)), and the great majority would recommend extending the MI intervention (somewhat yes: 42/320 (13.1%), yes: 261/320 (81.6%)) to other maternity wards. Almost all (308/320 (96.3%)) found the MI intervention useful (somewhat yes: 30/320 (9.4%), yes: 278/320 (86.9%)), and 90.3% (289/320) had no more questions about vaccination afterwards. Nearly the entire MI group (303/320 (94.7%)) considered that the intervention was performed in a timely manner (somewhat yes: 38/320 (11.9%), yes: 265/320 (82.8%)). In the control group, all participants (334/334) completed this part of the questionnaire, and satisfaction with the control intervention (leaflet) was similar, although the proportions of ‘somewhat yes’ answers were higher than in the MI group (see details of the control group results in Supplementary Material S8). Among MI participants specifically, 306/318 (96.2%) reported that it had respected their views on vaccination (somewhat yes: 24/318 (7.5%), yes: 282/318 (88.7%)) and 301/318 (94.7%) that its duration was appropriate. No participant in either group reported harm.

## Discussion

This study shows that an educational intervention on vaccination of newborns using MI with mothers in maternity wards in France was significantly associated with a 32.7% reduction in their VH score and an 8.5% increase in their intention to vaccinate their infants at 2 months of age. This was observed in a context where 11 childhood vaccines are required for admission to daycare and schools. Stratified analyses found the MI intervention's effect on VH and VI was greatest in mothers with a high initial VH level and differed only slightly by social deprivation level (measured through education level and perceived financial situation). The D-I-D analysis estimated the net decrease in VH due to the MI intervention at 5.8/100 points and the net increase in VI at 0.6/10 points. The D-I-D stratified analyses indicated a still higher net effect of MI on VH and VI in mothers with a higher initial VH level and revealed a differential effect by social deprivation level: the decrease in VH related to MI was only found in mothers with a low level of education, while the increase in VI was only found in mothers with a higher level of education. Most mothers in both groups were satisfied, although the control group had more ‘somewhat’ responses (24.6–31.1%, versus 9.4–17.5% in the MI group).

Comparisons with the Quebec study results show a similar magnitude of the MI intervention's effect on mothers’ VH. It was −33% in our study, −40% in the PromovaQ study, and −29% in the EMMIE programme [20,21]. The initial level of VH in the MI group was higher in our study (35/100) than in either of the Quebec studies (27/100 in PromoVaQ and 25/100 in EMMIE), an observation consistent with France and Canada’s VH rankings [36]. The 9% increase in mothers’ intention to vaccinate their infant at 2 months of age is again similar to that of the PromoVaQ study (12%) and the EMMIE programme (11%). As in the latter studies, our results suggest that the impact of the MI intervention is more marked among participants with greater initial VH.

Our study adds to the two aforementioned studies the use of D-I-D, to estimate the intervention's net effect compared with the control group. We found that MI's impact on both VH and VI remained significant, and at a relatively high level of magnitude compared with other educational approaches [12,37]. Moreover, a possible contamination effect in the control group (the midwives delivered the leaflet with some explanations about its content) might have led to underestimating this net effect.

Our results suggest that MI could reduce VH especially among mothers who are part of socially deprived populations, whose VH was highest in our study. The MI approach (listening, non-judgemental) probably enabled these mothers to better express their concerns and allowed midwives to respond more appropriately than a standardised leaflet could allow (no significant VH reduction found among the less educated in the control arm). This suggests that MI may be useful for addressing social inequalities in vaccine acceptance [4,5], provided it pays specific attention to using universally understandable language [29]. The experience of the Quebec EMMIE programme also highlights the importance of continuing to apply this intervention to all mothers, even those less hesitant [21]. Even mothers with little hesitation can have some concerns about vaccination, which MI allows them to express. Answering their questions may strengthen their confidence in vaccinating their infants, a useful effect in this era of vaccine misinformation.

Our results regarding mothers' satisfaction are similar to those found in the PromoVac study and EMMIE programme [15,21] and indicate adherence to MI's principles during the intervention. In particular, participants perceived that the midwives respected their autonomy; the latter thus demonstrated this MI skill essential for building trust. Participants' satisfaction of the MI during the maternity stay suggests that the intervention was well-accepted, despite the already substantial demands on mothers' time during this period. Furthermore, the performance of MI interventions complements the benefits of midwives' practice of empathetic listening to parents' concerns [38].

This study has several limitations. We measured intentions to vaccinate rather than actual vaccination status since childhood vaccines were almost all required at the time of survey and, as a result, vaccination rates were high, which would have prevented us from showing MI's effect, if any, on infant vaccine coverage. Moreover, 21% of the 257 women who refused participation were motivated by lack of interest in vaccination. Selection and/or reporting bias(es) leading to a false conclusion of a significant difference between the groups nonetheless seem(s) unlikely, as consent to participate was obtained prior to randomisation and few mothers refused to take part because of disinterest in vaccination (n = 53 mothers compared with 733 included). The short follow-up period at the hospital raises the possibility of a Hawthorne effect in both groups. Moreover, because the study could not be anonymised and the mothers spent 10–30 min talking with a midwife in the MI arm, a differential Hawthorne effect leading to an overestimation of MI's effect seems possible [31]. Nonetheless, the preliminary results of the assessment we conducted at 7 months, on average, after the birth indicate a net MI effect on VH of the same size as at T1 (data not shown) and suggest that a strong Hawthorne effect is unlikely. The initial VI score was slightly higher in the control than the MI group, which might have resulted in underestimating MI's net effect on VI. Fewer patients than anticipated were included, notably because of the COVID-19 pandemic; statistical power nonetheless remained sufficient to analyse the sample as a whole. The dropout rate between the first and second questionnaires was substantial at the Saint-Joseph maternity ward; however, the similar results of the ITT and PP analyses indicate that this did not affect the results. Finally, despite the universal nature of the MI principles, the generalisation of these results to other European countries requires caution, due to different socio-cultural contexts; the MI’s impact should therefore be assessed where childhood vaccination coverage is insufficient and/or where there is parental vaccine hesitancy.

The study has several strengths that minimise its potential biases. The similarity of demographic characteristics and initial VH scores between the two groups validates the randomisation process. The very low rate of secondary refusals (after randomisation) indicates participants' acceptance of this procedure. Finally, our protocol is close to that of the published Promovac and PromovaQ studies and EMMIE programme. In particular, we used the same validated instrument for the main judgement criteria (PACV) [15-21].

## Conclusion

The impact of MI on mothers’ VH and intentions to vaccinate, the apparent reduction of social disparities in vaccine acceptance and parental satisfaction with several aspects of the programme all argue for investigating conditions for larger-scale implementation of MI intervention in France possibly through an implementation study, including an assessment of its impact on vaccination delays between the recommended and actual age at vaccination and a cost-effectiveness analysis.

## Supplementary Data

676000 Supplement
