# Supplementary material for: A postpartum intervention for vaccination promotion by midwives using motivational interviews reduces mothers’ vaccine hesitancy, south-eastern France, 2021 to 2022: a randomised controlled trial
Source: Euro Surveill. 2023 Sep 21;28(38):2200819. doi: 10.2807/1560-7917.ES.2023.28.38.2200819 (PMC10515496; doi:10.2807/1560-7917.ES.2023.28.38.2200819)
Supplement: Supplement [file 22-00819_VERGER_SUPPLEMENT.pdf]

Supplementary material

This supplementary material is hosted by Eurosurveillance *as supporting information alongside the article "*Effectiveness of postpartum motivational interviews by midwives at the maternity ward to address parents' vaccine hesitancy: a randomized controlled trial in France in 2021-2022"*, on behalf of the authors, who remain responsible for the accuracy and appropriateness of the content. The same standards for ethics, copyright, attributions and permissions as for the article apply. Supplements are not edited by* Eurosurveillance *and the journal is not responsible for the maintenance of any links or email addresses provided therein.*

Supplementary Material S1. Adaptation of the Parents Attitudes about Childhood Vaccines (PACV) questionnaire

The PACV questionnaire has been validated to identify vaccine-hesitant parents and to predict future vaccine refusal (1–3) and has been translated and validated in French (4). As in the Promovac studies, two questions were removed for this study to adapt the questionnaire to the maternity ward setting ("*Have you ever delayed having your child get a shot for reasons other than illness or allergy?"; "Have you ever decided not to have your child get a shot for reasons other than illness or allergy?"*). The 13 retained items covered behaviours, general attitudes, and the perceived safety and efficacy of vaccines, with three categories of response reflecting hesitancy, non-hesitancy, or a '*not sure or don't know*' option.

Supplementary Material S2. Equation of the D-I-D model

The equation for the D-I-D model is as follows:

$$y_{it} = \beta_0 + \beta_{time} * Time_t + \beta_{group} * Group_i + \beta_{interaction} * Time_t * Group_i + \varepsilon_{it}$$

where y_{it} is the mean value of the expected outcome (VH score or intention to vaccinate one's infant) for patient *i* at time *t,*

$Time$ is a binary indicator that the "outcome" was measured in the pre- (0) or post-intervention period (1),

$Group$ is a binary indicator indicating if the patient is in the leaflet (0) or MI group (1)

and ε_{it} is the error term for the outcome measure of patient *i* at time *t*.

Parameters estimated are:

β_0, the pre-intervention average in the leaflet group (reference situation),

β_{time}, the time trend, i.e., the difference between the pre- and post-intervention periods, in the leaflet group,

β_{group}, the difference between the MI and leaflet groups in the pre-intervention period,

and $\beta_{interaction}$, the net difference in the changes over time between the two groups.

The mean value of the leaflet group in the pre-intervention period equals β_0, while the mean value of the MI group in the post-intervention period equals $\beta_0 + \beta_{time} + \beta_{group} + \beta_{interaction}$.

Supplementary Material S3. Distribution of the participants’ characteristics according to the two maternity wards

Table S1. Distribution of the participants’ characteristics according to the two maternity wards

Characteristics	Maternity ward				All		p value
	Sainte-Musse		Saint-Joseph				
	n = 416	%	n = 317	%	n = 733	%	
Age of the mother (years)							
All, mean (SD)	30.4 (5.0)		**31.7 (4.9)**		31.0 (5.0)		**0.001**
18–24	41	**9.9**	24	7.6	65	8.9	**0.05**
25–29	136	**32.7**	82	25.9	218	29.7	
30–34	152	36.5	122	**38.5**	274	37.4	
≥ 35	87	20.9	89	**28.1**	176	24.0	
Live with a partner[a]							
Yes	379	91.3	288	91.4	667	91.4	**0.01**
No	31	**7.5**	13	4.1	44	6.0	
Don't know/refuse to answer	5	1.2	14	**4.4**	19	2.6	
Birth rank of the newborn							
1	203	48.8	170	53.6	373	50.9	0.20
2 or more	213	51.2	147	46.4	360	49.1	
Education level[b]							
Equivalent to high school or lower	168	**40.5**	92	29.1	260	35.6	**0.001**
At least some post-secondary education	230	55.4	218	**69.0**	448	61.3	
Don't know/refuse to answer	17	**4.1**	6	1.9	23	3.2	
Perceived financial situation[a]							
Insecure	147	**35.4**	71	22.5	218	29.9	**0.0001**
Not insecure	250	60.2	216	**68.6**	466	63.8	
Don't know/refuse to answer	18	4.3	28	**8.9**	46	6.3	
Vaccinated against seasonal influenza during pregnancy[b]							
Yes	45	10.8	39	12.3	84	11.5	0.61
No	366	88.2	272	86.1	638	87.3	
Don't know/refuse to answer	4	1.0	5	1.6	9	1.2	
Vaccine hesitancy score							
Initial vaccine hesitancy score (0–100), mean (SD)	34.7 (20.9)		33.0 (20.1)		34.0 (20.6)		0.34
Vaccine intention score							
Initial score of intention to vaccinate one’s infant at 2 months of age[c] (1–10), mean (SD)	6.9 (13.0)		7.5 (10.6)		7.2 (12.0)		0.72
Agree to be contacted again for another questionnaire							
Yes	329	**79.1**	233	73.5	562	76.7	**0.0001**
No	87	20.9	71	**22.4**	158	21.6	
Don't know/refuse to answer	0	0.0	13	**4.1**	13	1.8	
**Randomisation group**							
MI-based intervention	207	49.8	156	49.2	363	49.5	0.88
Leaflet	209	50.2	161	50.8	370	50.5	
**Post-intervention questionnaire** (T1)							
Completed	409	**98.3**	247	77.9	656	89.5	**<.0001**
Missing	7	1.7	70	**22.1**	77	10.5	

MI: motivational interviewing; SD: standard deviation.

[a] Missing values (n = 3).

[b] Missing values (n = 2).

[c] Missing values (n = 11).

Because of rounding, the sum of the percentages may not equal 100%. Chi-squared tests were used for categorical variables and Wilcoxon rank sum tests were used for continuous variables.

An asterisk marks a p value ≤ 0.05, which was considered significant.

Supplementary Material S4. Overall difference in the pre-post VH score changes, in ITT (Table S2) and PP (Table S3) analyses

Table S2. Net difference in pre-post change of the VH score between the MI and control groups and factors associated with this score (D-I-D model, ITT, n=733)

	Without covariables (n=733)	With covariables (n=664)
	aβ [95% CI]	
Intercept (reference situation)	33.2 [31.1; 35.2] [a]	23.6 [19.1; 28.0]
Post-intervention period (ref. Pre-intervention)	**-5.5 [-8.5; -2.6]** [b]	**-6.0 [-8.9; -3.1]**
MI-based intervention group (ref. control)	1.6 [-1.3; 4.5] [c]	0.4 [-2.5; 3.3]
Net difference in pre-post change between the MI and control groups (ref. pre-intervention and control)	**-5.8 [-10.0; -1.6]** [d]	**-5.0 [-9.1; -0.8]**
Maternity ward (ref. Sainte Musse)		
St Joseph		0.6 [-1.6; 2.8][e]
Age (ref. 35 years and older)		
18-24	-	3.8 [-0.8; 8.3]
25-29	-	2.3 [-0.7; 5.3]
30-34	-	0.2 [-2.5; 2.9]
Birth rank of the newborn (ref. 2 or more)		
1	-	-0.3 [-2.6; 1.9]
Education level (ref. Post-secondary education)		
<= High school	-	**6.0 [3.6; 8.4]**
Perceived financial situation (ref. Insecure)		
Not insecure	-	**-6.9 [-9.2; -4.6]**
Vaccinated against influenza during pregnancy (ref. Yes)		
No	-	**12.4 [9.2; 15.6]**

* Reference situation: leaflet group at baseline; and, in the model including covariables, mothers aged ≥ 35 years, parous, education level at least some post-secondary education, feeling financially insecure, vaccinated against influenza during pregnancy.

Reading: [a] In the model without the covariables, the baseline average VH score of the control group was 33.2/100.

[b] This VH score significantly decreased by 5.5 points (95% confidence interval: -8.5; -2.6) after receiving the leaflet.

[C] At baseline, the VH score of the MI group was slightly but not significantly higher (+1.6 points [-1.3; 4.5]) than in the control group.

[d] The net impact of the MI intervention on the change in VH scores was significant (-5.8 points [-10.0; -1.6]); it is estimated by the interaction term Post-period * MI with the pre-period and control group as a reference.

[e] The analysis with the covariables shows a similar estimate of the net effect of the MI intervention (-5.0 [-9.1; -0.8]) and that there was no maternity ward effect (0.6 [-1.6; 2.8]).

Table S3. Net difference in pre-post change of the VH score between the MI and control groups and factors associated with this score (D-I-D model, PP, n=656)

	Without covariables (n=656)	With covariables (n=598)
	aβ [95% CI]	
Intercept (reference situation)	32.8 [30.6;34.9] [a]	23.1 [18.6;27.6]
Post-intervention period (ref. Pre-intervention)	**-5.1 [-8.1 ;-2.1]** [b]	**-5.6 [-8.6;-2.6]**
MI group (ref. leaflet)	1.9 [-1.1;4.9] [c]	1.0 [-2.0;4.0]
Post- period * MI (ref. pre-intervention and/or leaflet)	**-6.2 [-10.4;-1.9]** [d]	**-5.6 [-9.8;-1.3]**
Maternity ward (ref. Sainte Musse)		
St Joseph		0.1 [-2.2; 2.3] [e]
Age (ref. 35 years and older)		
18-24	-	4.1 [-0.6; 8.7]
25-29	-	2.0 [-1.1; 5.0]
30-34	-	0.5 [-2.3; 3.3]
Birth rank of the newborn (ref. 2 or more)		
1	-	0.0 [-2.3; 2.3]
Education level (ref. Post-secondary education)		
<= High school	-	**5.9 [3.5; 8.4]**
Perceived financial situation (ref. Insecure)		
Not insecure	-	**-6.8 [-9.1; -4.4]**
Vaccinated against influenza during pregnancy (ref. Yes)		
No	-	**12.4 [9.1; 15.7]**

* Reference situation: leaflet group at baseline; and, in the model including covariables, mothers aged ≥ 35 years, parous, education level at least some post-secondary education, feeling financially insecure, vaccinated against influenza during pregnancy.

[a] In the model without the covariables, the baseline average VH score of the control group was 32.8/100.

[b] This VH score significantly decreased by 5.1 points (95% confidence interval: -8.1; -2.1) after receiving the leaflet.

[C] At baseline, the VH score of the MI group was slightly, but not significantly, higher (+1.9 points [-1.1; 4.9]) than in the control group.

[d] The net impact of the MI intervention on the change in VH scores was significant (-6.2 points [-10.4; -1.9]); it is estimated by the interaction term Post-period * MI with the pre-period and control group as a reference.

[e] The analysis with the covariables shows a similar estimation of the net effect of the MI (-5.6 [-9.8; -1.3]) and that there was no maternity ward effect (0.1 [-2.2; 2.3]).

Supplementary Material S5. Overall difference in pre-post changes of vaccination intentions, in ITT (Table S4) and PP (Table S5)

Table S4. Net difference in pre-post change of the score of vaccination intentions regarding participants' infants at 2 months of age between the MI and control groups and factors associated with this score (D-I-D model, ITT, n=733)

	Without covariables (n=733)	With covariables (n=664)
	aβ [95% CI]	
Intercept (reference situation)	8.7 [8.5; 8.8] [a]	8.9 [8.5; 9.4]
Post-intervention period (ref. Pre-intervention)	0.1 [-0.1; 0.4] [b]	0.1 [-0.2; 0.4]
MI group (ref. leaflet)	**-0.3 [-0.6; -0.1]** [c]	**-0.3 [-0.6; -0.05]**
Post-period * MI (ref. pre-intervention and/or leaflet)	**0.6 [0.2; 0.9]** [d]	**0.6 [0.2; 1.0]**
Maternity ward (ref. Sainte Musse)		
St Joseph	-	-0.2 [-0.4; 0.0] [e]
Age (ref. 35 years and older)	-	
18-24	-	-0.1 [-0.5; 0.3]
25-29		-0.1 [-0.4; 0.2]
30-34	-	0.1 [-0.2; 0.3]
Birth rank of the newborn (ref. 2 or more)		
1	-	0.1 [-0.1; 0.3]
Education level (ref. Post-secondary education)		
<= High school	-	-0.1 [-0.3; 0.2]
Perceived financial situation (ref. Insecure)		
Not insecure	-	**0.6 [0.3; 0.8]**
Vaccinated against influenza during pregnancy (ref. Yes)		
No	-	**-0.6 [-1.0; -0.3]**

* Reference situation: leaflet group at baseline; and, in the model including covariables, mothers aged ≥ 35 years, parous, education level at least some post-secondary education, feeling financially insecure, vaccinated against influenza during pregnancy.

[a] In the model without the covariables, the mean baseline score of intention to vaccinate their infant at 2 months of age (VI) in the control group was 8.7/10.

[b] This VI score increased slightly, but not significantly, by 0.1 point (95% confidence interval: -0.1; 0.4) after receiving the leaflet.

[C] At baseline, the VI score of the MI group was significantly lower (-0.3 points [-0.6; -0.1]) than in the control group.

[d] The net impact of the MI intervention on the change in VI scores was significant (+0.6 points [0.2; 0.9]): it is estimated by the interaction term Post-period * MI taking the pre-period and control group as a reference.

[e] The analysis with the covariables shows a similar estimation of the net effect of the MI intervention (0.6 [0.2; 1.0]) and that there was no maternity ward effect (-0.2 [-0.4; 0.0]).

Table S5. Net difference in pre-post change of the score of vaccination intentions regarding participants' infants at 2 months of age between the MI and control groups and factors associated with this score (D-I-D model, PP, n=656)

	Without covariables (n=656)	With covariables (n=598)
	aβ [95% CI]	
Intercept (reference situation)	8.7 [8.5; 8.9] [a]	9.0 [8.5; 9.4]
Post-intervention period (ref. Pre-intervention)	0.2 [-0.1; 0.4] [b]	0.1 [-0.2; 0.4]
MI-based intervention group (ref. leaflet)	**-0.4 [-0.7; -0.1]** [c]	**-0.4 [-0.7; -0.1]**
Post-period * MI (ref. pre-intervention and/or leaflet)	**0.6 [0.2; 1.0]** [d]	**0.6 [0.2; 1.0]**
Maternity ward (ref. Sainte Musse)		
St Joseph	-	-0.1 [-0.3; 0.2] [e]
Age (ref. 35 years and older)	-	
18-24	-	-0.1 [-0.5; 0.3]
25-29		-0.2 [-0.5; 0.1]
30-34	-	0.0 [-0.3; 0.2]
Birth rank of the newborn (ref. 2 or more)		
1	-	0.0 [-0.2; 0.3]
Education level (ref. Post-secondary education)		
<= High school	-	-0.1 [-0.3; 0.2]
Perceived financial situation (ref. Insecure)		
Not insecure	-	**0.5 [0.3; 0.8]**
Vaccinated against influenza during pregnancy (ref. Yes)		
No	-	**-0.6 [-0.9; -0.3]**

* Reference situation: leaflet group at baseline; and, in the model including covariables, mothers aged ≥ 35 years, parous, education level at least some post-secondary education, feeling financially insecure, vaccinated against influenza during pregnancy.

Reading: [a] In the model without the covariables, the mean baseline average intention to vaccinate their infant at 2 months of age (VI) score of the control group was 8.7/10.

[b] This VI score increased slightly but not significantly, by 0.2 point (95% confidence interval: -0.1; 0.4) after receiving the leaflet.

[C] At baseline, the VI score of the MI group was significantly lower (-0.4 points [-0.7; -0.1]) than in the control group.

[d] The net impact of the MI intervention on the change in VI scores was significant (+0.6 points [0.2; 1.0]); it is estimated by the interaction term Post-period * MI with the pre-period and control group as a reference.

[e] The analysis with the covariables shows a similar estimate of the net effect of MI intervention (0.6 [0.2; 1.0]) and that there was no maternity ward effect (-0.1 [-0.3; 0.2]).

Supplementary Material S6. Difference in pre-post VH score changes after stratification for perceived financial situation

Analyses stratified for perceived financial situation showed that VH pre-scores were around 10 percentage points (pp) higher among participants feeling insecure than among those feeling financially not insecure, in both the MI (40.4% *vs* 30.4%) and leaflet (38.6% *vs* 30.4%) groups (Figure S1). In the MI group, in the post-intervention period, VH decreased significantly among mothers feeling either insecure (-14.1 pp, from 40.4% to 26.3%: 34.9% decrease, p<0.0001) or not (-9.6 pp, from 30.4% to 20.8%: 31.5% decrease, p<0.0001) (Figure 1). In the leaflet group, the VH score significantly decreased only among participants who felt not insecure (from 30.4% to 23.8%: 21.8% decrease, p=0.0002); the decrease among those who felt insecure (from 38.6% to 34.7%: 10.1% decrease, p=0.16) was not significant. The D-I-D effect of MI compared with the leaflet on VH scores was significant among participants feeling financially insecure (-10.2 pp, p=0.001) but not among those not insecure (-2.9 pp, p=0.26).

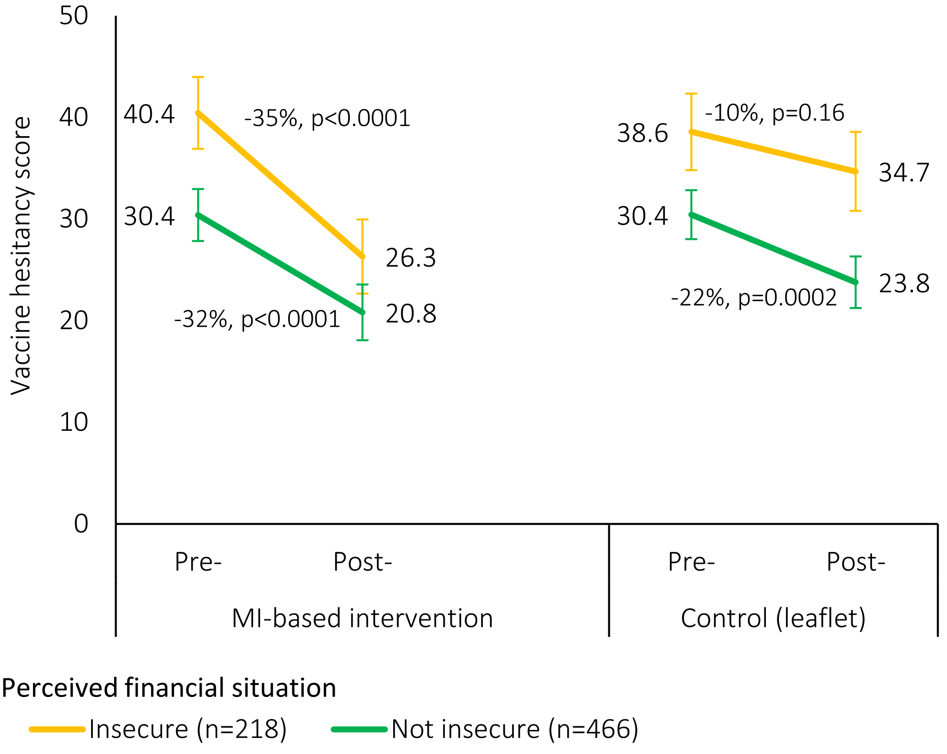

Figure S1. Description of changes in vaccine hesitancy scores by intervention group according to perceived financial situation, south-eastern France, November 2021- April 2022 (n = 684[a])

D-I-D: difference-in-difference; MI: Motivational interview; VH: Vaccine hesitancy.

Scores for VH were estimated with 95% confidence intervals in the D-I-D model, at T0 and T1, according to group (MI or control) and perceived financial situation (insecure: n = 116 (MI) and 102 (control); not insecure: n = 219 (MI) and 247 (control)).

[a] Some participants (n = 46) did not know about their financial situation or refused to answer, or data were missing (n = 3).

Note: Among the participants feeling insecure, VH scores in the MI group decreased by 34.7%, from 40.4% to 26.3% (p < 0.0001).

Supplementary Material S7. Difference in pre-post changes in vaccination intentions after stratification for perceived financial situation

Analyses stratified for the perceived financial situation showed that vaccination intention pre-scores were lower among participants feeling insecure than among those feeling financially not insecure, in both the MI (7.8 *vs* 8.6/10) and leaflet (8.5 *vs* 8.9/10) groups (Figure S2). In the MI group, post-intervention, vaccination intentions increased significantly among mothers, either insecure (+0.9 points, from 7.8 to 8.7/10: 12.0% increase, p=0.001) or not (+0.6 points, from 8.6 to 9.2/10: 6.5% increase, p=0.001). In the leaflet group, there was no significant change among participants according to their perceived financial situation (insecure: from 8.5 to 8.3, p=0.48; not insecure: from 8.9 to 8.8/10, p=0.69). The D-I-D effect of MI versus the leaflet on vaccination intention scores was significant among participants feeling financially not insecure (+0.5 points, p=0.03) but not among those feeling insecure (+0.7 points, p=0.07).

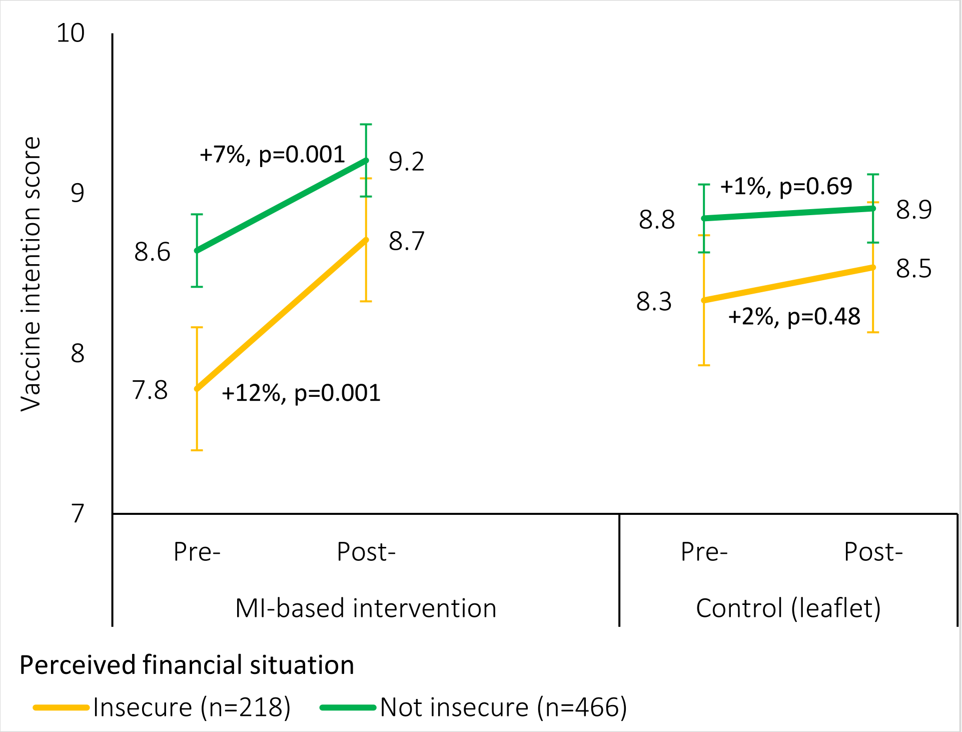

Figure S2. Descriptions of changes in vaccine intention scores by intervention group according to perceived financial situation, south-eastern France, November 2021- April 2022 (n = 684[a])

D-I-D: difference-in-difference; MI: Motivational interview; VH: Vaccine hesitancy.

Vaccination intention scores were estimated with their 95% confidence intervals in the D-I-D model, at T0 and T1, by group (MI or control) and perceived financial situation (insecure: n = 116 (MI) and 102 (control); not insecure: n = 219 (MI) and 247 (control)).

[a] Some participants (n = 46) did not know about their financial situation or refused to answer, or data were missing (n = 3).

Note: Among participants feeling insecure, vaccination intention scores in the MI group increased by 12.0%, from 7.8 to 8.7/10 (p=0.001).

Supplementary Material S8. Control group satisfaction results

In the control group, 334/334 mothers answered the satisfaction questionnaire: 311/334 (93.1%) reported having appreciated participating (somewhat yes: 126/334 (37.7%); yes: 185/334 (55.4%)), and the great majority would recommend the extension of the leaflet (somewhat yes: 82/334 (24.6%), yes: 216/334 (64.7%)) to other maternity units. Nine mothers of ten had found the leaflet useful (somewhat yes: 104/334 (31.1%), yes: 196/334 (58.7%)), and 264/334 (79.0%) had no more questions about vaccination afterwards.

Supplementary Material S9: Questionnaires

PRE-QUESTIONNAIRE T0 before intervention/leaflet (English version)

1. Do you know which diseases are prevented by the vaccines offered to your baby between 2 and 6 months?
□ Not at all
□ Somewhat
□ Quite well
□ Very well
□ Don't know
□ Do not wish to answer

2. Do you know which vaccines are available for your baby to prevent these diseases?
□ Not at all
□ Somewhat
□ Quite well
□ Very well
□ Don't know
□ Do not wish to answer

For the following statements, indicate whether you strongly disagree, disagree, agree or strongly agree.

3. I feel sufficiently informed to make a decision about vaccinating my baby.
□ Strongly disagree
□ Disagree
□ Neither agree nor disagree
□ Agree
□ Strongly agree
□ Do not wish to answer

4. It is important to start my baby's vaccinations at 2 months.
□ Strongly disagree
□ Disagree
□ Neither agree nor disagree
□ Agree
□ Strongly agree
□ Do not wish to answer

5. If my baby gets a disease that I didn't vaccinate against, I will regret it.
□ Strongly disagree
□ Disagree
□ Neither agree nor disagree
□ Agree
□ Strongly agree
□ Do not wish to answer

6. Before I make a decision about vaccinating my baby, I will get detailed information.
□ Strongly disagree
□ Disagree
□ Neither agree nor disagree
□ Agree
□ Strongly agree
□ *Do not wish to answer*

7. In deciding whether to vaccinate your baby, are the opinions of the following people and information sources important to you?

	Not at all important	Not very important	Neither important nor not important	Somewhat important	Very important	Do not wish to answer
Spouse	□	□	□	□	□	□
Family	□	□	□	□	□	□
Friends	□	□	□	□	□	□
Your doctor (general practitioner or paediatrician)	□	□	□	□	□	□
A professional practising alternative medicine (naturopath, homeopath, chiropractor, etc.)	□	□	□	□	□	□
Information produced by the Ministry of Health	□	□	□	□	□	□
The Vaccination Info Services website	□	□	□	□	□	□
Social networks (Facebook, etc.)	□	□	□	□	□	□

7.BIS. Is any other source of information important to you?
□ Yes
□ No
□ *Do not wish to answer*
If "yes": Specify which one: ....

8. Are you not insecure with having to vaccinate your child?
□ Not at all
□ Somewhat
□ Quite
□ Very
□ *Don't know*
□ *Do not wish to answer*

9. Do you know where to go to get your baby vaccinated?
□ Yes
□ No
□ *Do not wish to answer*

10. Do you know who will follow up your baby (paediatrician, family doctor, etc.)?
□ Yes
□ No
□ *Do not wish to answer*

11. Where do you think you will have your baby vaccinated?
□ Your family doctor
□ At a paediatrician
□ At a Maternal and Child Protection Service office
□ Other
□ I do not know
□ I will not vaccinate my baby
□ *Do not wish to answer*

If 'Other': Where else would you consider vaccinating your baby?

....

12. Thinking about vaccination in general, would you say that you personally are...
Circle a number between 0 and 10, where 0 is very unfavourable to vaccination and 10 is very favourable to vaccination

Very unfavourable to vaccination										Very favourable to vaccination
0	1	2	3	4	5	6	7	8	9	10

□ *Do not wish to answer*

13. Do you want your baby to get all the recommended shots?[1]
□ Yes
□ No
□ *Don't know*
□ *Do not wish to answer*

14. Overall, how hesitant about childhood shots would you consider yourself to be?
□ Not at all hesitant
□ Not too hesitant
□ Neither hesitant nor not hesitant
□ Somewhat hesitant
□ Very hesitant
□ *Do not wish to answer*

For the following statements, indicate whether you strongly disagree, disagree, agree or strongly agree.

[1] Items 13 to 25 have been adapted from the Parents' Attitudes about Childhood Vaccines (PACV) questionnaire. Opel DJ, Taylor JA, Mangione-Smith R, Solomon C, Zhao C, Catz S, et al. Validity and reliability of a survey to identify vaccine-hesitant parents. Vaccine. 2011;29(38):6598–605.

15. Children get more shots than are good for them.
□ Strongly disagree
□ Disagree
□ Neither agree nor disagree
□ Agree
□ Strongly agree
□ *Do not wish to answer*

16. I believe that many of the illnesses shots prevent are severe.
□ Strongly disagree
□ Disagree
□ Neither agree nor disagree
□ Agree
□ Strongly agree
□ *Do not wish to answer*

17. It is better for my child to develop immunity by getting sick than to get a shot.
□ Strongly disagree
□ Disagree
□ Neither agree nor disagree
□ Agree
□ Strongly agree
□ *Do not wish to answer*

18. It is better for children to get one vaccine at a time.
□ Strongly disagree
□ Disagree
□ Neither agree nor disagree
□ Agree
□ Strongly agree
□ *Do not wish to answer*

19. I trust the information I receive about shots.
□ Strongly disagree
□ Disagree
□ Neither agree nor disagree
□ Agree
□ Strongly agree
□ *Do not wish to answer*

20. I am able to openly discuss my concerns about shots with my child's doctor.
□ Strongly disagree
□ Disagree
□ Neither agree nor disagree
□ Agree
□ Strongly agree
□ *Do not wish to answer*

21. How concerned are you that your child might have a serious side effect from a shot?
□ Not at all concerned
□ Not too concerned
□ Neither concerned nor unconcerned
□ Somewhat concerned
□ Very concerned
□ *Do not wish to answer*

22. How concerned are you that any one of the childhood shots might not be safe?
□ Not at all concerned
□ Not too concerned
□ Neither concerned nor unconcerned
□ Somewhat concerned
□ Very concerned
□ *Do not wish to answer*

23. How concerned are you that a shot might not prevent the disease?
□ Not at all concerned
□ Not too concerned
□ Neither concerned nor unconcerned
□ Somewhat concerned
□ Very concerned
□ *Do not wish to answer*

24. How sure are you that following the recommended shot schedule is a good idea for your child?
Circle a number between 1 and 10, where 1 is not at all sure and 10 is completely sure that the recommended shot schedule is a good idea for your child.

Not at all sure — Completely sure

1 2 3 4 5 6 7 8 9 10

□ *Do not wish to answer*

25. All things considered, how much do you trust your child's doctor?
Circle a number between 1 and 10, where 1 is not to trust at all and 10 is to trust your child's doctor completely.

Do not trust at all — Completely trust

1 2 3 4 5 6 7 8 9 10

□ *Do not wish to answer*

26. How sure are you to vaccinate your baby at 2 months of age?
Circle a number between 1 and 10, where 1 is not at all sure and 10 is completely sure about having your baby vaccinated at 2 months.

Not at all sure — Completely sure

1 2 3 4 5 6 7 8 9 10

□ *Do not wish to answer*

27. Are you feeling stress about your baby's vaccination?
Circle a number between 0 and 10, where 0 is no stress and 10 is a lot of stress about your baby's vaccination.

No stress — A lot of stress

0 1 2 3 4 5 6 7 8 9 10

□ *Do not wish to answer*

28. Do you feel that you have little or no control over your baby's vaccination?
Circle a number between 0 and 10, where 0 is feeling that you have absolutely no control and 10 is feeling that you have total control over your baby's vaccination.

Absolutely no control										Total control
0	1	2	3	4	5	6	7	8	9	10

□ *Do not wish to answer*

29. What is your current age?
__________ years old
□ *Do not wish to answer*

30. What is the highest degree you have obtained?
□ No diploma
□ CEP, brevet des collèges, CAP, BEP
□ Baccalauréat *(examination at the end of high school)*
□ 1st cycle university diploma (licence, BTS...)
□ 2nd cycle university diploma or more (master, doctorate...)
□ *Don't know*
□ *Do not wish to answer*

31. Were you vaccinated against flu during your pregnancy?
□ Yes
□ No
□ *Don't know*
□ *Do not wish to answer*

32. Do you live with a partner or spouse?
□ Yes
□ No
□ *Don't know*
□ *Do not wish to answer*

33. At present, in your household, financially, would you say that...
□ You can't manage without going into debt
□ You can't manage easily
□ You have to be careful
□ It's only fair
□ It's OK
□ You are not insecure
□ *Don't know*
□ *Do not wish to answer*

We thank you for your valuable collaboration, and you can be assured of the confidentiality of the data collected.

POST-QUESTIONNAIRE T1 after motivational interview or leaflet (English version)

Only if post-MI questionnaire

0. Participation of the spouse in the interview:

□ Yes
□ No

The following questions are common to both the post-MI and post-leaflet questionnaires, unless specified

1. Do you know which diseases are prevented by the vaccines offered to your baby between 2 and 6 months?

□ Not at all
□ Somewhat
□ Quite well
□ Very well
□ *Don't know*
□ *Do not wish to answer*

2. Do you know which vaccines are available for your baby to prevent these diseases?

□ Not at all
□ Somewhat
□ Quite well
□ Very well
□ *Don't know*
□ *Do not wish to answer*

For the following statements, indicate whether you strongly disagree, disagree, agree or strongly agree.

3. I feel sufficiently informed to make a decision about vaccinating my baby.

□ Strongly disagree
□ Disagree
□ Neither agree nor disagree
□ Agree
□ Strongly agree
□ *Do not wish to answer*

4. It is important to start my baby's vaccination at 2 months.

□ Strongly disagree
□ Disagree
□ Neither agree nor disagree
□ Agree
□ Strongly agree
□ *Do not wish to answer*

5. If my baby gets a disease that I didn't vaccinate against, I will regret it.
□ Strongly disagree
□ Disagree
□ Neither agree nor disagree
□ Agree
□ Strongly agree
□ *Do not wish to answer*

6. Are you not insecure with having to vaccinate your child?
□ Not at all
□ Somewhat
□ Quite
□ Very
□ *Don't know*
□ *Do not wish to answer*

7. Do you know where to go to get your baby vaccinated?
□ Yes
□ No
□ *Do not wish to answer*

8. Do you know who will follow up your baby (paediatrician, family doctor, etc.)?
□ Yes
□ No
□ *Do not wish to answer*

9. Where do you think you will have your baby vaccinated?
□ Your family doctor
□ At a paediatrician
□ At a Maternal and Child Protection Service office
□ Other
□ I do not know
□ I will not vaccinate my baby
□ *Do not wish to answer*

If 'Other': Where else would you consider vaccinating your baby?

....

10. Thinking about vaccination in general, would you say that you personally are...
Circle a number between 0 and 10, where 0 is very unfavourable to vaccination and 10 is very favourable to vaccination

Very unfavourable to vaccination — Very favourable to vaccination

0 1 2 3 4 5 6 7 8 9 10

□ *Do not wish to answer*

11. Do you want your baby to get all the recommended shots?[2]
□ Yes
□ No
□ *Don't know*
□ *Do not wish to answer*

12. Overall, how hesitant about childhood shots would you consider yourself to be?
□ Not at all hesitant
□ Not too hesitant
□ Neither hesitant nor not hesitant
□ Somewhat hesitant
□ Very hesitant
□ *Do not wish to answer*

For the following statements, indicate whether you strongly disagree, disagree, agree or strongly agree.

13. Children get more shots than are good for them.
□ Strongly disagree
□ Disagree
□ Neither agree nor disagree
□ Agree
□ Strongly agree
□ *Do not wish to answer*

14. I believe that many of the illnesses shots prevent are severe.
□ Strongly disagree
□ Disagree
□ Neither agree nor disagree
□ Agree
□ Strongly agree
□ *Do not wish to answer*

15. It is better for my child to develop immunity by getting sick than to get a shot.
□ Strongly disagree
□ Disagree
□ Neither agree nor disagree
□ Agree
□ Strongly agree
□ *Do not wish to answer*

16. It is better for children to get one vaccine at a time.
□ Strongly disagree
□ Disagree
□ Neither agree nor disagree
□ Agree
□ Strongly agree
□ *Do not wish to answer*

---

[2] Items 11 to 23 have been adapted from the Parents' Attitudes about Childhood Vaccines (PACV) questionnaire. Opel DJ, Taylor JA, Mangione-Smith R, Solomon C, Zhao C, Catz S, et al. Validity and reliability of a survey to identify vaccine-hesitant parents. Vaccine. 2011;29(38):6598–605.

17. I trust the information I receive about shots.
□ Strongly disagree
□ Disagree
□ Neither agree nor disagree
□ Agree
□ Strongly agree
□ *Do not wish to answer*

18. I am able to openly discuss my concerns about shots with my child's doctor.
□ Strongly disagree
□ Disagree
□ Neither agree nor disagree
□ Agree
□ Strongly agree
□ *Do not wish to answer*

19. How concerned are you that your child might have a serious side effect from a shot?
□ Not at all concerned
□ Not too concerned
□ Neither concerned nor unconcerned
□ Somewhat concerned
□ Very concerned
□ *Do not wish to answer*

20. How concerned are you that any one of the childhood shots might not be safe?
□ Not at all concerned
□ Not too concerned
□ Neither concerned nor unconcerned
□ Somewhat concerned
□ Very concerned
□ *Do not wish to answer*

21. How concerned are you that a shot might not prevent the disease?
□ Not at all concerned
□ Not too concerned
□ Neither concerned nor unconcerned
□ Somewhat concerned
□ Very concerned
□ *Do not wish to answer*

22. How sure are you that following the recommended shot schedule is a good idea for your child?
Circle a number between 1 and 10, where 1 is not at all sure and 10 is completely sure that the recommended shot schedule is a good idea for your child.

Not at all sure									Completely sure
1	2	3	4	5	6	7	8	9	10

□ *Do not wish to answer*

23. All things considered, how much do you trust your child's doctor?
Circle a number between 1 and 10, where 1 is not to trust at all and 10 is to trust your child's doctor completely.

Do not trust at all									Completely trust
1	2	3	4	5	6	7	8	9	10

□ *Do not wish to answer*

24. How sure are you to vaccinate your baby at 2 months of age?
Circle a number between 1 and 10, where 1 is not at all sure and 10 is completely sure about having your baby vaccinated at 2 months.

Not at all sure									Completely sure
1	2	3	4	5	6	7	8	9	10

□ *Do not wish to answer*

25. Are you feeling stress about your baby's vaccination?
Circle a number between 0 and 10, where 0 is no stress and 10 is a lot of stress about your baby's vaccination.

No stress										A lot of stress
0	1	2	3	4	5	6	7	8	9	10

□ *Do not wish to answer*

26. Do you feel that you have little or no control over your baby's vaccination?
Circle a number between 0 and 10, where 0 is feeling that you have absolutely no control and 10 is feeling that you have total control over your baby's vaccination.

Absolutely no control										Total control
0	1	2	3	4	5	6	7	8	9	10

□ *Do not wish to answer*

Satisfaction questionnaire

27.
If post-MI questionnaire: **Did you enjoy participating in this interview?**
If post-leaflet questionnaire: **Did you appreciate the leaflet you were given about childhood immunisation?**

□ No
□ Lean no
□ Neither yes nor no
□ Lean yes
□ Yes
□ *Do not wish to answer*

If 'No' or 'Lean No': Why? ....
□ *Don't know*
□ *Do not wish to answer*

28.
If post-MI questionnaire: **Would you recommend that this type of interview be offered to other parents in maternity wards?**
If post-leaflet questionnaire: **Would you recommend this leaflet to other parents in maternity wards?**
□ No
□ Lean no
□ Neither yes nor no
□ Lean yes
□ Yes
□ *Do not wish to answer*
If 'No' or 'Lean No': Why? ....
□ *Don't know*
□ *Do not wish to answer*

Only if post-MI questionnaire
29. Do you feel that the interview respected your views on vaccination?
□ No
□ Lean no
□ Neither yes nor no
□ Lean yes
□ Yes
□ *Do not wish to answer*
If 'No' or 'Lean No': Why? ....
□ *Don't know*
□ *Do not wish to answer*

30.
If post-MI questionnaire: **Did you find it useful to discuss vaccination with the provider?**
If post-leaflet questionnaire: **Did you find this leaflet useful?**
□ No
□ Lean no
□ Neither yes nor no
□ Lean yes
□ Yes
□ *Do not wish to answer*
If 'No' or 'Lean No': Why? ....
□ *Don't know*
□ *Do not wish to answer*

31.
If post-MI questionnaire: **Did the timing of the interview suit you?**
If post-leaflet questionnaire: **Did the timing of the handover suit you?**
□ No
□ Lean no
□ Neither yes nor no
□ Lean yes
□ Yes
□ *Do not wish to answer*
If 'No' or 'Lean No': Why? ....
□ *Don't know*
□ *Do not wish to answer*

Only if post-MI questionnaire
32. Was the length of the interview...
□ Too long
□ Slightly too long
□ Neither too long nor too short
□ Slightly too short
□ Too short
□ *Do not wish to answer*

33.
If post-MI questionnaire: **Following this interview, do you still have questions about vaccination?**
If post-leaflet questionnaire: **After reading this leaflet, do you have any questions about vaccination?**
□ Yes
□ No
□ *Don't know*
□ *Do not wish to answer*

If "Yes": Which ones? ....
....
....
....
....
....
....
....

□ *Don't know*
□ *Do not wish to answer*

34.
If post-MI questionnaire: **Do you have any comments or suggestions for the interview?**
If post-leaflet questionnaire: **Do you have any comments or suggestions for the leaflet?**
....
....
....
....
....
....
....
....
....

We thank you for your valuable collaboration, and you can be assured of the confidentiality of the data collected.

References

1. Opel DJ, Mangione-Smith R, Taylor JA, Korfiatis C, Wiese C, Catz S, et al. Development of a survey to identify vaccine-hesitant parents: The parent attitudes about childhood vaccines survey. Hum Vaccin. 2011 Apr;7(4):419–25.
2. Opel DJ, Taylor JA, Zhou C, Catz S, Myaing M, Mangione-Smith R. The relationship between parent attitudes about childhood vaccines survey scores and future child immunization status: a validation study. JAMA Pediatr. 2013 Nov;167(11):1065-71.
3. Opel DJ, Taylor JA, Mangione-Smith R, Solomon C, Zhao C, Catz S, et al. Validity and reliability of a survey to identify vaccine-hesitant parents. Vaccine. 2011 Sep 2;29(38):6598–605.
4. Olarewaju VO, Jafflin K, Deml MJ, Zimmermann C, Sonderegger J, Preda T, et al. Application of the Parent Attitudes about Childhood Vaccines (PACV) survey in three national languages in Switzerland: Exploratory factor analysis and Mokken scale analysis. Hum Vaccin Immunother. 2021 Aug 3;17(8):2652-2660.
